# Predictors of Recurrent Ureteral Stones: A Retrospective Analysis of Clinical, Demographic, and Metabolic Factors

**DOI:** 10.7759/cureus.104093

**Published:** 2026-02-23

**Authors:** Mostafa Arafa, Karim Farhat, Farrukh K Khan, Abdulaziz M Althunayan, Mohamed F Farahat, Mashael Alshebaili, Danny Rabah

**Affiliations:** 1 Department of Surgery, The Cancer Research Chair, College of Medicine, King Saud University, Riyadh, SAU; 2 Department of Epidemiology, High Institute of Public Health, Alexandria, EGY; 3 Department of Surgery, College of Medicine, King Saud University, Riyadh, SAU; 4 Department of Community Health Sciences, College of Applied Medical Sciences, King Saud University, Riyadh, SAU; 5 Department of Obstetrics and Gynecology, College of Medicine, King Saud University, Riyadh, SAU; 6 Department of Urology, King Faisal Specialist Hospital and Research Centre, Riyadh, SAU

**Keywords:** bmi, gout, metabolic disorders, recurrence, ureteral stones, uric acid stones

## Abstract

Background

While global meta-analyses have identified broad risk factors for kidney stone recurrence, data on specific determinants of recurrent ureteral stones in the Middle Eastern region remain limited. We aimed to identify the clinical, demographic, and metabolic determinants of recurrent ureteral stones, including body mass index (BMI).

Materials

This retrospective cohort study was conducted at a single institution in Saudi Arabia. Clinical data from 57 patients with ureteral stones were analyzed. The variables included age, sex, BMI, stone composition, comorbidities (diabetes mellitus (DM), hypertension (HT), and gout), and back pain. Univariate (independent t-tests and chi-square tests) and multivariate logistic regression models were used to assess associations with recurrence. Recurrence was defined as two or more episodes of stone passage or intervention within 12 months.

Results

Recurrence occurred in 30% of the patients. On multivariate analysis, gout (odds ratio (OR): 8.6, 95% confidence interval (CI): 1.3-56.7, p = 0.026) and uric acid stones (OR: 4.9, 95% CI: 1.1-21.9, p = 0.038) were independently associated with recurrence, while back pain showed a trend toward significance (OR: 4.1, 95% CI: 0.9-18.6, p = 0.07). BMI, age, and sex were not significantly associated (p > 0.05).

Conclusion

Gout and uric acid stone composition were independent predictors of ureteral stone recurrence. Despite the limitations of a small sample size and short follow-up period, targeted metabolic screening and management of hyperuricemia may reduce the risk of recurrence.

## Introduction

Ureteral stones affect 1-3% of the global population, with recurrence rates reaching up to 50% within 10 years [1,2]. Globally, calcium oxalate stones are the most prevalent (70-80%), while uric acid stones account for 5-10% of cases [3]. However, regional variations are significant; in Saudi Arabia, the prevalence of urolithiasis is among the highest in the world, reported at up to 20.1% [4]. Local studies indicate that while calcium oxalate remains the most common composition (67.8-76%), the prevalence of uric acid stones is notably higher in Saudi Arabian cohorts (up to 19.7%) compared to Western populations [5]. Recurrence is driven by a complex interplay between metabolic abnormalities (e.g., hyperuricemia and hypercalciuria), dietary habits, and anatomical factors [6]. Obesity, measured by body mass index (BMI), is a recognized risk factor for kidney stone formation owing to its association with insulin resistance and metabolic syndrome [7]. However, its specific role in ureteral stone recurrence remains poorly understood, particularly in Middle Eastern populations, where the prevalence of obesity is increasing [8]. This study aimed to identify the clinical, demographic, and metabolic predictors of recurrent ureteral stones, with a focus on the role of BMI. Previous research has linked recurrence to comorbidities such as diabetes mellitus (DM), hypertension (HT), and gout [9, 10]. Uric acid stones are particularly prone to recurrence without targeted therapy because of acidic urine pH and hyperuricemia [11]. By evaluating these factors in a cohort from Saudi Arabia, this study provides novel insights into the predictors of ureteral stone recurrence and the potential influence of BMI.

## Materials and methods

Inclusion and exclusion criteria

The inclusion criteria were patients with complete clinical data (57 cases). These 57 cases represented all eligible patients with complete clinical and metabolic data from the parent study during the specified period. The exclusion criteria were patients with incomplete clinical data.

Study design, location, and duration of study

This is a retrospective cohort study. The study was conducted at King Saud University Medical City in Riyadh, Saudi Arabia, from January 2022 to 2024.

Study tools

This study was conducted using the electronic health records of patients diagnosed with ureteral stones who were previously enrolled in a larger prospective randomized study at the urology outpatient clinic at King Saud University Medical City, Riyadh, Saudi Arabia [12]. To handle overlap, only patients with complete follow-up and metabolic data relevant to recurrence were included in this retrospective sub-analysis. Data were extracted using standardized forms and included demographic variables such as age, sex, height, and weight. Clinical variables were elaborated to include stone characteristics (laterality and composition), comorbidities (DM, HT, and gout), history of back pain, and medication history (including the use of thiazide diuretics). Additionally, BMI was calculated as weight (kg) divided by height squared (m²) and categorized as normal (<25 kg/m²), overweight (25-29.9 kg/m²), or obese (≥30 kg/m²). Height and weight were measured by trained nursing staff at the time of admission using standardized equipment. Comorbidities were defined as follows: DM (fasting blood glucose level ≥126 mg/dL or use of antidiabetic medication), HT (systolic blood pressure ≥140 mmHg or use of antihypertensive medication), and gout (clinical diagnosis or use of urate-lowering therapy). Comorbidities were assessed at baseline (initial presentation). The stone composition was determined by infrared spectroscopy analysis of the passed or surgically retrieved stones when available. Due to the retrospective nature of the study and variations in clinical practice, comprehensive spectroscopic analysis and 24-hour urine metabolic testing were not uniformly available for all patients. Therefore, stone composition and metabolic data were included where available, and stones were broadly categorized as uric acid stones or non-uric acid stones. The stone laterality was recorded as unilateral or bilateral. For bilateral cases, the largest stone or stone requiring intervention was used for the primary analysis. Back pain was recorded as a binary variable (present/absent) based on patient self-reports or physician documentation of pain requiring analgesic intervention at the time of initial stone presentation only. Recurrence was defined as two or more episodes of ureteral stone passage or intervention (e.g., lithotripsy or ureteroscopy) within 12 months. This 12-month definition was chosen to identify early recurrence and high-risk metabolic stone formers, as utilized in previous prospective studies [13].

Approval was granted by the Institutional Review Board (IRB) of the College of Medicine, King Saud University (Date: 01.04.2015/No: 15/0127/IRB).

Statistical tests

Descriptive statistics were calculated as follows: continuous variables were presented as mean ± standard deviation (SD) and categorical variables as percentages. The normality of continuous variables (age and BMI) was assessed using the Shapiro-Wilk test. As the data followed a normal distribution, independent t-tests were used to compare means between the recurrence and no-recurrence groups. For categorical variables, chi-square tests were used, and Fisher’s exact test was applied for small cell counts (expected frequency < 5). Missing data were handled by excluding cases with incomplete records, as specified in the exclusion criteria. Multivariate logistic regression, performed using the Statistical Package for the Social Sciences (SPSS) version 26 (IBM Corp., Armonk, NY), was used to assess the independent predictors of recurrence, with recurrence as the dependent variable and age, sex, BMI, stone composition, comorbidities, and back pain as independent variables. Multicollinearity among independent variables was assessed using the variance inflation factor (VIF), with a VIF < 5 considered acceptable. Given the limited sample size and number of recurrence events (n = 17), the number of variables in the final multivariate model was restricted to those with a p-value < 0.1 in univariate analysis to maintain an adequate events-per-variable ratio. The significance threshold was set at p < 0.05.

## Results

Baseline characteristics

Of the 57 patients, 30% (17) experienced recurrence. The mean age was 44.3 ± 12.1 years, and 65% were male. The BMI distribution within the cohort indicated that 47% (27) of patients had a normal BMI, 35% (20) were overweight, and 18% (10) were obese. Uric acid stones were present in 28% of the cases (Table 1). The recurrence rates across BMI categories were 26%, 35%, and 30% for normal-weight, overweight, and obese patients, respectively.

**Table 1:** Baseline characteristics

Variable	Recurrence (n = 17)	No recurrence (n = 40)
Age (years)	46.1 ± 13.2	43.5 ± 11.7
Male	11 (65%)	26 (65%)
Female	6 (35%)	14 (35%)
BMI (kg/m²)	26.8 ± 4.1	25.9 ± 4.3
DM	5 (29%)	12 (30%)
HT	6 (35%)	14 (35%)
Uric acid stones	9 (53%)	6 (15%)
Gout	5 (29%)	2 (5%)
Back pain	15 (88%)	30 (75%)

Univariate analysis

Univariate analysis revealed no significant association between recurrence and age (p = 0.45), sex (p = 0.99), BMI (p = 0.42), DM (p = 0.78), or HT (p = 0.65). Medication history, including the use of thiazide diuretics, was also analyzed; however, due to the low prevalence of use in this cohort (7%), no significant association with recurrence was observed (p > 0.05). Significant associations were observed for uric acid stones (χ² = 6.2, p = 0.013), gout (χ² = 5.8, p = 0.016), and back pain (χ² = 2.8, p = 0.094) (Table 2).

**Table 2:** Univariate logistic regression analysis for predictors of ureter stone recurrence For categorical variables (sex, DM, HT, uric acid stones, gout, and back pain), the chi-square test was used. The t-test was used for continuous variables (age and BMI). DM, diabetes mellitus; HT, hypertension

Variable	Statistics	p-value
Age	t = 0.76	0.45
Sex	χ²	0.99
BMI	t = 0.81	0.42
DM	χ²	0.78
HT	χ²	0.65
Uric acid stones	χ²	0.013
Gout	χ²	0.016
Back pain	χ²	0.094

Multivariate analysis

In the multivariate model adjusted for age, sex, and BMI, gout (OR: 8.6, 95% CI: 1.3-56.7, p = 0.026) and uric acid stones (OR: 4.9, 95% CI: 1.1-21.9, p = 0.038) were independently associated with recurrence. Back pain showed a trend toward significance (OR: 4.1, 95% CI: 0.9-18.6, p = 0.07). BMI remained non-significant (p = 0.42) (Table 3).

**Table 3:** Multivariate logistic regression analysis for predictors of ureter stone recurrence The model was adjusted for age, sex, and body mass index (BMI). BMI was not significantly associated with recurrence (p = 0.42).

Variable	Odds ratio (95% CI)	p-value
Gout	8.6 (1.3-56.7)	0.026
Uric acid stones	4.9 (1.1-21.9)	0.038
Back pain	4.1 (0.9-18.6)	0.07

## Discussion

This study identified gout and uric acid stone composition as independent predictors of recurrent ureteral stones, reinforcing the link between metabolic disorders and urolithiasis [14,15]. The association between gout and recurrence highlights the role of hyperuricemia in stone formation. Hyperuricemia lowers urinary pH, increases oxalate excretion, and reduces citrate levels, fostering a lithogenic environment [16]. These findings align with those of studies reporting a two- to three-fold higher recurrence risk in patients with gout [10,17]. Current guidelines from the European Association of Urology (EAU) and the American Urological Association (AUA) recommend basic metabolic testing, including serum uric acid, for all stone formers [18]. Despite these recommendations, clinical compliance remains low, with some studies reporting that 24-hour urine testing is completed in less than 10% of high-risk patients [19]. Routine assessment of serum uric acid is particularly valuable, as hyperuricemia has been shown to promote the formation of both uric acid and calcium-based stones [20]. 

The independent association between uric acid stones and recurrence reflects their solubility profiles. Unlike calcium oxalate stones, uric acid stones may grow undetected in acidic urine, leading to larger burdens and higher recurrence rates [21]. A meta-analysis by Sakhaee et al. [22] noted that uric acid stones, although only 5-10% of all stones, carry a disproportionately high risk of recurrence, particularly in patients with metabolic syndrome. In our cohort, 53% of recurrent cases had uric acid stones versus 15% of non-recurrent cases, suggesting that targeted interventions such as urine alkalinization (pH 6.2-6.8) or allopurinol could reduce recurrence [23].

BMI was not significantly associated with recurrence (p = 0.42) despite its established role in stone formation [7]. Obesity promotes uric acid stones via insulin resistance and acidic urine [3], with each 5 kg/m² BMI increasing stone risk by 30-50% [23]. The lack of significance may stem from the small sample size or unmeasured factors, such as visceral adiposity, which is a stronger lithogenesis predictor than BMI [16]. Future studies should explore body composition and metabolic markers (e.g., insulin resistance) to clarify the role of BMI.

Limitations

The findings of this study must be interpreted cautiously owing to several significant methodological limitations. First, the small sample size (N = 57) and low number of recurrence events (n = 17) severely limited the statistical power of the multivariate logistic regression model. This is evidenced by the extremely wide 95% CIs for the significant predictors (e.g., gout OR: 1.3-56.7), which indicate high uncertainty in the effect estimates and limit the generalizability of the magnitude of the ORs. Second, the definition of recurrence as two or more episodes within a short 12-month period is a major limitation. Urolithiasis recurrence is typically defined over a much longer period (e.g., five to 10 years) to capture true metabolic new stone formation. The 12-month window may predominantly capture events related to residual fragments from the initial stone episode or immediate post-treatment complications rather than new stone formation driven by underlying metabolic risk factors. This short follow-up period may have biased the results and limited their clinical relevance to long-term preventive strategies. Third, the inclusion of non-specific symptoms, such as back pain, is problematic since it lacks clinical specificity. While the trend toward significance (p = 0.07) may suggest an association with larger stones, delayed diagnosis, or residual fragments post-intervention [24], these underlying factors should ideally be measured and controlled directly. The current use of a nonspecific proxy introduced a significant confounding variable.

## Conclusions

This retrospective cohort study identified gout and uric acid stone composition as significant independent predictors of recurrent ureteral stones, with ORs of 8.6 and 4.9, respectively. These findings underscore the critical role of metabolic disorders, particularly hyperuricemia, in the pathogenesis of recurrent urolithiasis. Despite the limitations of a small sample size and short 12-month follow-up period, the results advocate routine metabolic screening, including serum uric acid levels and 24-hour urine analysis, in patients with recurrent ureteral stones. The observed gap in routine adherence to metabolic screening guidelines, as noted in our study, highlights a critical area for improvement in clinical practice. Early identification of hyperuricemia or gout could enable targeted interventions, such as urine alkalinization or xanthine oxidase inhibitors, to mitigate the risk of recurrence. Future research should focus on prospective, larger-scale studies with longer follow-up periods to validate these findings and explore the nuanced roles of BMI and other metabolic syndrome components in stone recurrence.
